# Timed up and go test predicts mortality in older adults in Peru: a population-based cohort study

**DOI:** 10.1186/s12877-022-02749-6

**Published:** 2022-01-18

**Authors:** Edson J. Ascencio, Gustavo D. Cieza-Gómez, Rodrigo M. Carrillo-Larco, Pedro J. Ortiz

**Affiliations:** 1 School of Medicine, Universidad Peruana Cayetano Heredia, Lima, Peru; 2 CRONICAS Centre of Excellence in Chronic Diseases, Universidad Peruana Cayetano Heredia, Lima, Peru; 3 Department of Epidemiology and Biostatistics, School of Public Health, Imperial College London, London, UK; 4 Gerontology Institute, Universidad Peruana Cayetano Heredia, Av. Honorio Delgado 430, San Martín de Porres, Lima, Peru

**Keywords:** Physical function, Timed up and go test, Mortality, Global health, Risk assessment

## Abstract

**Background:**

While there is evidence about stablished risk factors (e.g., raised blood pressure) and higher mortality risk in older population, less has been explored about other functional parameters like the Timed Up and Go test and the Gait Speed in older people at low- and middle-income countries. We aimed to study these mobility tests as predictors of mortality in a population of older people in Peru.

**Methods:**

Population-based prospective cohort study (2013–2020). Random sampling of people aged 60+ years in a community of Lima, Peru. Geriatricians conducted all clinical evaluations and laboratory tests were conducted in the local hospital. Participants were sought in the national vital registration system, and we collated cause (ICD-10) and date of death. We conducted a nested forward multivariate Cox proportional hazard model to identify all potential predictors of all-cause, communicable and non-communicable diseases mortality.

**Results:**

At baseline, there were 501 older people (mean age 70.6 and 62.8% were women), complete follow-up information was available from 427 people. Mean follow-up time was 46.5 months (SD = 25.3). In multivariate models, the Timed Up and Go test was associated with higher risk of all-cause mortality (HR = 1.05; 95% CI: 1.02–1.09). For cause-specific mortality, history of heart disease (HR = 2.25; 95% CI: 1.07–4.76) and age in years (HR = 1.05; 95% CI: 1.01–1.09) were predictors of non-communicable diseases mortality.

**Conclusions:**

In addition to established risk factors for mortality in older population, the Timed Up and Go test, a functional parameter, raised as a relevant predictor of all-cause mortality.

**Supplementary Information:**

The online version contains supplementary material available at 10.1186/s12877-022-02749-6.

## Introduction

Aging of the population is a phenomenon fast occurring in most countries. This has sparked interest in finding risk factors for death as a result of deviations of homeostatic equilibrium, deteriorations of health and multimorbidity, while considering the complexity and heterogeneity of older adults [1]. Among other risk factors, poor mobility is of great relevance and goes beyond the overall health status of the older adult affecting their independent and quality of life [2–5].

Mobility problems in older people can be detected with tests such as the Timed Up and Go test [6] and Gait Speed [7]. These are useful to predict falls [8] and to determine frailty older people [9]. Moreover, these tests are independently associated with a higher risk of mortality [7, 10–12], including mortality due to Non-Communicable Diseases (NCD) both in the presence of comorbidities and in the absence of known cardiovascular risk factors [11, 12]. Whether this mortality risk holds in all older populations, particularly those in low- and middle-income countries in Latin America, where prevalence of NCD, distribution of cardiovascular risk factors and access to medical care are different than in high-income countries [13, 14], is unknown.

In contrast to high-income countries, high prevalence of mental and chronic diseases, mainly dementia, frailty, depression and disability [15–19] directly increase rates of well-known associated adverse outcomes including mortality. Also, older people in LMIC had lower rates of socioeconomic support and health insurance [20–22], leading to inequity, a low level of health services access and high out-of-pocket expenses to attend their health needs [23, 24]. Finally, guidelines to risk assessment for older people could not be followed, as in other frequent conditions as diabetes and hypertension [25, 26], mainly because they are not flexible, adaptable, sociocultural accepted and economically attainable.

Even the aforementioned differences in older people’s characteristics of vulnerability between high-income versus LMIC and the well described role of Timed Up and Go test and Gait Speed in predict mortality in high-income countries [5, 27–29], scarce studies are done in LMIC and mainly related with all-cause mortality [30]. This evidence gap prevents recommending the application of mobility tests as a structural part of the geriatric evaluation [31]. Considering the Timed Up and Go test has the ability to explore the interactions in different systems like cardiopulmonary, nervous and musculoskeletal systems involved in it, we hypothesized that Timed Up and Go test is a good predictor not only for all-cause mortality, but also for NCD and non-NCD mortality.

To provide evidence to strengthen the recommendation of including mobility tests as part of the regular geriatric consultation in low- and middle-income countries particularly those in Latin America, we aimed to determine if mobility tests, such as Timed Up and Go test and Gait Speed, are independent predictors of mortality (all-causes, NCD, and due to infectious diseases/accidents), in a population-based cohort of community-dwelling older adults in Lima, Peru.

## Methods

### Study design

Originally, this was a cross-sectional study conducted in 2013 [19]. We turned this cross-sectional study into a prospective cohort by looking for the original participants in the vital registration system on 8th March 2020; from the vital registration system, we retrieved survival status (dead or alive), date of death and the underlying cause of death (ICD-10 code). These codes are found in an Additional file (see Additional file 1). We adhered to the Strengthening the Reporting of Observational Studies in Epidemiology (STROBE) guidelines [32] and the study was approved by the Institutional Ethics Committee for Humans at Cayetano Heredia University in Lima, Peru (Reference number: 207–06-20).

### Study setting

Peru is a middle-income country located in South America. This study was developed in *San Martin de Porres*, the second most populated district in Lima, which is the capital of Peru. This district has a current population of 755,087 residents and 10.9% of them live in poverty [33]. Period of baseline recruitment was from January to May 2013 and the survival status was ascertained on 8th March 2020. Data collection was performed by four geriatricians through a face-to-face interview at baseline. They applied a structured questionnaire that registered demographics, socioeconomic information, and a comprehensive geriatric evaluation.

### Participants

Adults older than 60 years who lived in the district of *San Martín de Porres* were included. A total of 501 participants were enrolled and evaluated with a health interview (e.g., self-reported diseases), a physical examination (e.g., weight and height), and laboratory tests (e.g., total cholesterol).

We followed a semi-probabilistic sampling of household clusters. *San Martin de Porres* was divided into eight sections, according to the distribution of sixteen primary healthcare centres and one hospital. In each section, blocks were given a random number. Every day the research team was assigned one block, until the sample size for each section was reached. In each block, households were randomly selected. In each household, all those aged 60+ years were selected; if there were no older people living in the selected household, the adjacent home was visited.

Further details about the sampling methods and procedures of the cohort are available elsewhere [16, 19, 34]. On 8th March 2020, the national vital registration system was queried to ascertain the vital status of the participants, and when applicable, date and cause of death were retrieved as well.

### Variables

#### Predictors

Participants self-reported the following information (self-reported diseases and habits): heart diseases, stroke, rheumatology diseases, respiratory diseases, tuberculosis, edentulous, insomnia, visual impairment, hearing impairment, falls, incontinence, polypharmacy and tobacco consumption.

For this analysis, diabetes mellitus, hypertension, and dyslipidaemia were determined both by self-reported information and laboratory tests. We considered these variables as self-reported if an older adult had a previous diagnosis of each disease or if they reported the use of oral hypoglycaemic drugs or insulin for diabetes mellitus, antihypertensive drugs for hypertension, and statins, fibrates or ezetimibe for dyslipidaemia. Furthermore, as part of laboratory assessment, we considered diabetes mellitus with fasting glucose ≥126 mg/dL; hypertension with ≥140 mmHg or ≥ 90 mmHg for systolic and diastolic blood pressure, respectively; and (any) dyslipidaemia as total cholesterol ≥200 mg/dL, or LDL-cholesterol ≥130 mg/dL, or triglycerides ≥200 mg/dL, or HDL-cholesterol ≥50 mg/dL if the participant was female and HDL-cholesterol ≥40 mg/dL if the participant was male. The abdominal perimeter was measured in the physical examination. In the analysis, obesity was based on body mass index (BMI ≥30 kg/m^2^) computed from measured weight and height.

Validated scales were used to determine geriatric syndromes: Pfeiffer test for Cognitive evaluation [35], Yesavage Geriatric Depression Scale for Depression [36], Barthel Index for the functional status [37], Gijon Socio-familial Evaluation Scale for social evaluation [38], malnutrition was evaluated with the Mini-Nutritional Assessment [39] and family APGAR test (adaptability, partnership, growth, affection and resolve) to determine familial dysfunction [40]. All of these instruments are validated, has a good reliability and are used as part of the Comprehensive Geriatric Assessment in Peru [41].

Functional parameters were collected using the Gait Speed test and a Timed Up and Go test; both measurements were taken using a calibrated stopwatch. Gait Speed was determined by the time required for the participant to walk 8 m out of a total distance of 10 m at the normal walking speed, without a warm-up period. The first and last meter of the walk were not considered. The shorter time between two measurements was recorded. We set two Gait Speed thresholds (1 and 1.2 m/second) based on prospective studies of cardiovascular events [42].

The Timed Up and Go test was assessed with the older adult sat in a chair, asked to get up, walk 3 m, turn and return to the chair. It initiates by a cue from the measurer to get up from the chair and the measurer assessed the time it takes to sit down again. We set two Timed Up and Go test thresholds (10 and 15 s) according to previously published evidence [29, 43].

### Outcomes

The outcome was the survival status of the participants until 8th March 2020. We also studied cause-specific mortality in two groups: non-communicable diseases (NCDs) as well as accidents and infectious diseases. The ICD-10 codes in each group are shown in an Additional file (see Additional file 1).

### Study sample size

At baseline, we recruited a total of 501 participants in previous studies [16, 19, 34]. Based on that information, we calculated post-hoc sample size of 314 participants (with a power of 95%).

### Statistical analysis

Analyses were conducted with STATA SE 16.1 (StataCorp, College Station, TX, US). The statistical analysis code is available upon reasonable request.

First, characteristics of the study population were summarized using means and standard deviations (SD) or median and interquartile range (IQR) for numeric variables, depending on their distribution. Skewness and Kurtosis tests and histogram were used to assess the distribution. We did not categorize quantitative variables. To compare differences between outcome groups (alive vs death or NCDs vs accidents and infectious diseases) we used the Chi-squared or Fisher’s exact tests for categorical variables; and T-test or Mann-Whitney U tests for numerical variables. Second, survival rates were analysed using the Kaplan-Meier method and the differences between groups were analysed by the log-rank test. Third, univariate analysis was performed to identify prognostic variables related to overall survival. We adjusted the Model 1 with age, sex and educational level. A fully adjusted model (Model 2) was developed with a nested forward multivariate Cox proportional hazard regression approach, considering only univariate variables with *p* values < 0.05. Complete-case analysis was performed throughout this work.

## Results

### Study population

**Fig. 1:**
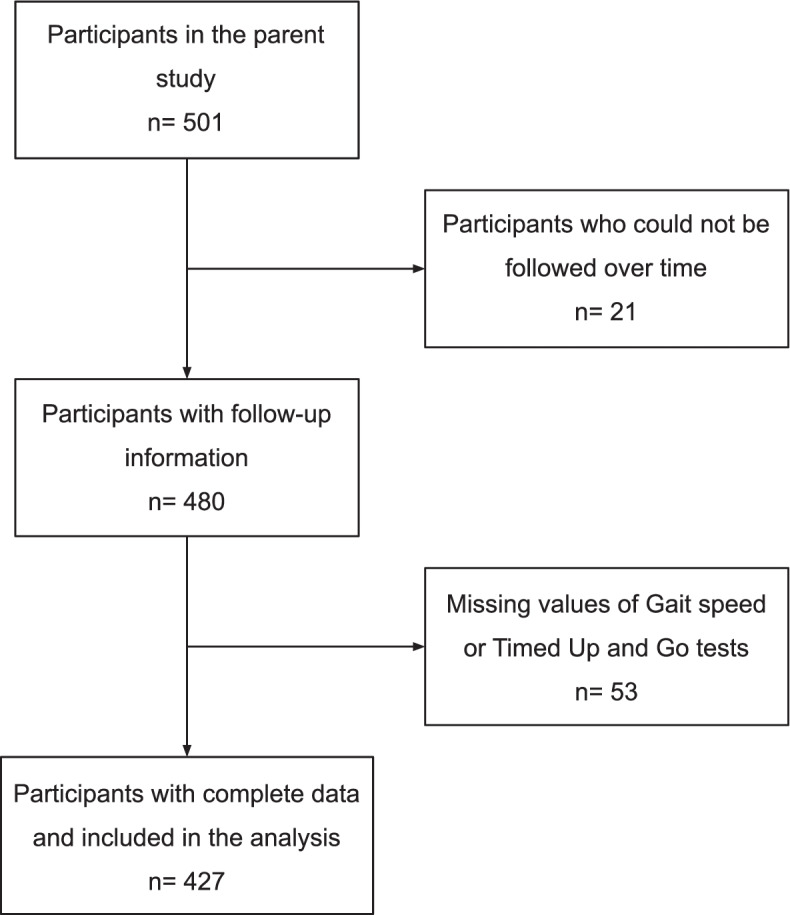
Flowchart of the population included in the analysis

As showed in Fig. 1, at baseline, there were 501 people, and follow-up information was available from 480 individuals; finally, 427 people with complete information in all variables of interest were herein analysed (89% of the study population with follow-up data).

There were more women (62.8%), and the overall mean age was 70.6 (standard deviation: 8.5) years at baseline; most of the study population had either primary (39.6%) or secondary (35.8%) education, while 19.2% had higher education at baseline. Overall, the mean Gait Speed was 1.00 (standard deviation: 0.3) meters/second; similarly, the median Timed Up and Go test was 10 (interquartile range: 9–13) seconds (Table 1).

**Table 1:** General characteristics of the study population

Characteristic	Survived *N* = 346	Dead (all-cause) *N* = 81	*p*-value	Dead by Infections or Accidents *N* = 43	Dead by NCD *N* = 38	*p*-value
**Gender**						
Male	117 (33.82%)	42 (51.85%)	0.003^b^	22 (51.16%)	20 (52.63%)	0.895^b^
Female	229 (66.18%)	39 (48.15%)		21 (48.84%)	18 (47.37%)	
Age (years); Median (IQR)	67 (63–74)	76 (68–83)	< 0.001^e^	78 (69–83)	75.5 (67–84)	0.336^e^
**Civil Status**						
Married or Cohabitation	219 (63.29%)	53 (65.43%)	0.719^b^	27 (62.79%)	26 (68.42%)	0.595^b^
Single, Divorced or Widower	127 (36.71%)	28 (34.57%)		16 (37.21%)	12 (31.58%)	
**Education**						
Higher	68 (19.65%)	14 (17.28%)	0.200^b^	6 (13.95%)	8 (21.05%)	0.235^c^
Secondary	130 (37.57%)	23 (28.40%)		9 (20.93%)	14 (36.84%)	
Primary	132 (38.15%)	37 (45.68%)		23 (53.49%)	14 (36.84%)	
No Education	16 (4.62%)	7 (8.64%)		5 (11.63%)	2 (5.26%)	
**Functional Parameters**						
Gait Speed (m/s); Mean ± SD	1.03 ± 0.25	0.87 ± 0.34	< 0.001^d^	0.81 ± 0.32	0.94 ± 0.35	0.081^d^
Timed Up and Go test (sec); Median (IQR)	10 (9–12)	12 (10–16)	< 0.001^e^	13 (10–18)	10.85 (9–15)	0.037^e^
**Clinical Variables**						
Abdominal perimeter (centimeters); Median (IQR)	98.75 (91–105)	100 (94–107)	0.169^e^	98 (91–106)	101 (97–108)	0.191^e^
Weight (kilograms); Median (IQR)	65 (56–74)	65 (55.5–73)	0.741^e^	62.5 (55–70)	66.8 (57–75.5)	0.168^e^
Malnutrition	94 (27.17%)	18 (22.22%)	0.362^b^	10 (23.26%)	8 (21.05%)	0.812^b^
Obesity	245 (70.81%)	56 (69.14%)	0.766^b^	25 (58.14%)	31 (81.58%)	0.023^b^
Dyslipidemia	214 (61.85%)	46 (56.79%)	0.401^b^	24 (55.81%)	22 (57.89%)	0.850^b^
Diabetes Mellitus^a^	62 (17.92%)	13 (16.05%)	0.691^b^	7 (16.28%)	6 (15.79%)	0.952^b^
Arterial Hypertension^a^	169 (48.84%)	51 (62.96%)	0.022^b^	25 (58.14%)	26 (68.42%)	0.339^b^
Heart Diseases^a^	25 (7.23%)	13 (16.05%)	0.012^b^	5 (11.63%)	8 (21.05%)	0.249^b^
Stroke^a^	12 (3.47%)	1 (1.23%)	0.477^c^	0 (0.00%)	1 (2.63%)	0.284^c^
Rheumatological Diseases^a^	128 (36.99%)	21 (25.93%)	0.060^b^	11 (25.58%)	10 (26.32%)	0.940^b^
Respiratory Diseases^a^	18 (5.20%)	8 (9.88%)	0.123^c^	5 (11.63%)	3 (7.89%)	0.574^c^
Tuberculosis^a^	5 (1.45%)	3 (3.70%)	0.179^c^	2 (4.65%)	1 (2.63%)	1.000^c^
Familial dysfunction^a^	74 (21.39%)	21 (25.93%)	0.377^b^	16 (37.21%)	5 (13.16%)	0.014^b^
Sociofamily impairment	204 (58.96%)	58 (71.60%)	0.035^b^	34 (79.07%)	24 (63.16%)	0.113^b^
Cognitive impairment	35 (10.12%)	18 (22.22%)	0.003^b^	7 (16.28%)	11 (28.95%)	0.171^b^
Insomnia^a^	249 (71.97%)	63 (77.78%)	0.288^b^	33 (76.74%)	30 (78.95%)	0.812^b^
Depression	66 (19.08%)	15 (18.52%)	0.908^b^	9 (20.93%)	6 (15.79%)	0.552^b^
Falls	113 (32.66%)	31 (38.27%)	0.336^b^	19 (44.19%)	12 (31.58%)	0.244^b^
Edentulous	294 (84.97%)	76 (93.83%)	0.035^b^	41 (95.35%)	35 (92.11%)	0.661^c^
Incontinence^a^	80 (23.12%)	19 (23.46%)	0.949^b^	11 (25.58%)	8 (21.05%)	0.631^b^
Visual problem	259 (74.86%)	62 (76.54%)	0.752^b^	31 (72.09%)	31 (81.58%)	0.315^b^
Hearing problem	139 (40.17%)	41 (50.62%)	0.087^b^	20 (46.51%)	21 (55.26%)	0.432^b^
Polypharmacy	135 (39.02%)	39 (48.15%)	0.132^b^	24 (55.81%)	15 (39.47%)	0.142^b^
Tobacco Consumption	32 (9.25%)	6 (7.41%)	0.828^b^	1 (2.33%)	5 (13.16%)	0.094^c^

*NCD* non-communicable disease), *IQR* Interquartile range, *SD* standard deviation

^a^ Diseases included in the table refer to self-reported history

^b^ calculated with Chi-squared test

^c^ calculated whit Fisher’s exact test

^d^ calculated with T-test

^e^ calculated with Mann-Whitney U test

### All-cause mortality

**Fig. 2:**
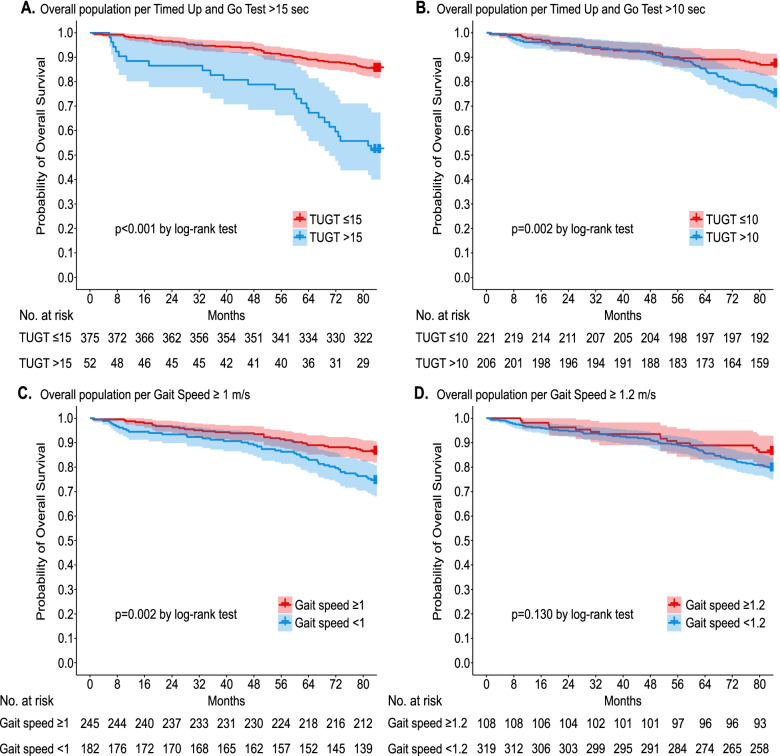
Survival curves (Kaplan-Meier) of cumulative unadjusted survival rates for the Timed Up and Go test and Gait Speed. Timed Up and Go test with a threshold of 15 s (**A**), for the Timed Up and Go test with a threshold of 10 s (**B**), for Gait Speed with a threshold of 1.0 m/second (**C**), and for the Gait Speed with a threshold of 1.2 m/second (**D**)

For all-cause mortality, the mean follow-up was 46.5 months (standard deviation: 25.3). The median Timed Up and Go test were longer among those who died (12 vs 10 s; *p* < 0.001) versus to those who survived (Table 1). We further stratified the Timed Up and Go test with thresholds at 15 and 10 s (Fig. 2A and B, respectively). In both cases, the survival decreased faster among those with longer Timed Up and Go test, and much faster when the Timed Up and Go test was set at > 15 s (Fig. 2A). Cumulative survival rates in Fig. 2 are unadjusted. The fully adjusted model revealed that the Timed Up and Go test increased the risk of all-cause mortality by 0.05 per one-unit change in seconds (1.05, 95% CI: 1.02–1.09; Table 2).

**Table 2:** Risk factors for all-cause mortality (*N* = 427)

Characteristic	Crude Model cHR (95% CI)	*p*-value	Model 1 aHR (95% CI)	*p*-value	Model 2 aHR (95% CI)	*p*-value
**Gender**						
Male	1		1		1	
Female	0.51 (0.33–0.78)	0.002	0.50 (0.32–0.79)	0.003	**0.52 (0.33–0.80)**	**0.003**
Age (years)	1.08 (1.06–1.10)	< 0.001	1.06 (1.03–1.08)	< 0.001	**1.06 (1.04–1.08)**	**< 0.001**
**Civil Status**						
Married or Cohabitation	1					
Single, Divorced or Widower	0.92 (0.58–1.46)	0.728				
**Education**						
Higher	1		1			
Secondary	0.84 (0.43–1.64)	0.605	0.68 (0.34–1.37)	0.281		
Primary	1.28 (0.68–2.40)	0.439	0.88 (0.46–1.66)	0.687		
No Education	1.83 (0.74–4.49)	0.188	1.04 (0.34–3.11)	0.950		
**Functional Parameters**						
Gait Speed (m/s)	0.15 (0.06–0.37)	< 0.001				
Timed Up and Go test (sec)	1.06 (1.03–1.11)	< 0.001	1.06 (1.02–1.09)	0.001	**1.05 (1.02–1.09)**	**0.001**
**Clinical Variables**						
Abdominal perimeter	1.00 (0.99–1.02)	0.434				
Weight	0.99 (0.98–1.01)	0.736				
Malnutrition	0.80 (0.47–1.35)	0.399				
Obesity	0.91 (0.57–1.47)	0.704				
Dyslipidemia^a^	0.82 (0.53–1.28)	0.384				
Diabetes Mellitus^a^	0.87 (0.48–1.55)	0.628				
Arterial Hypertension^a^	1.65 (1.05–2.60)	0.030			1.43 (0.91–2.25)	0.117
Heart Diseases^a^	2.16 (1.20–3.90)	0.010				
Stroke^a^	0.38 (0.05–2.77)	0.338				
Rheumatological Diseases^a^	0.64 (0.39–1.05)	0.079				
Respiratory Diseases^a^	1.87 (0.89–3.94)	0.097				
Tuberculosis^a^	2.27 (0.72–7.16)	0.161				
Familial dysfunction^a^	1.28 (0.78–2.11)	0.334				
Sociofamily impairment	1.62 (0.99–2.64)	0.053				
Cognitive impairment	2.12 (1.28–3.51)	0.004				
Insomnia^a^	1.28 (0.75–2.19)	0.357				
Depression	0.96 (0.55–1.69)	0.897				
Falls	1.24 (0.80–1.95)	0.338				
Edentulous	2.47 (0.99–6.17)	0.053				
Incontinence^a^	1.04 (0.62–1.75)	0.878				
Visual problem	1.05 (0.62–1.77)	0.859				
Hearing problem	1.43 (0.93–2.21)	0.107				
Polypharmacy	1.40 (0.90–2.16)	0.133				
Tobacco Consumption	0.78 (0.35–1.75)	< 0.548				

Model 1 adjusted variables: age, sex, educational level and Timed Up and Go Test. Model 2 was developed with a nested forward multivariate Cox proportional hazard regression. cHR (crude hazard ratio), aHR (adjusted hazard ratio), 95% CI (95% confidence interval)

^a^Diseases included in the table refer to self-reported history. Estimates in bold are statistically significant at *p* < 0.05

The mean Gait Speed was slower in older people who died (1.03 vs 0.87 m/seconds; *p* < 0.001) versus those who survived (Table 1). We further stratified the Gait Speed with thresholds at 1.0 and 1.2 m/second (Fig. 2C and D, respectively). With the first cut-off point (1.0 m/second), the survival rate decreased faster among those who died (Fig. 2C); conversely, the survival rate did not decrease faster when the cut-off point was set at 1.2 m/second (Fig. 2D). The Gait Speed was not included in the adjusted Cox models; the crude analysis suggested that faster Gait Speed would be associated with lower all-cause mortality risk (HR = 0.15, 95% CI: 0.06–0.37; Table 2).

### Cause-specific mortality

For NCDs mortality, the mean follow-up was 45.8 months (standard deviation: 26.4); for mortality due to accidents and infectious diseases the mean follow-up was 47.1 months (standard deviation: 24.7). The Timed Up and Go test was slower in older people who died of an infectious disease or an accident than in those who died of a NCD (13.0 vs 10.9 s; Table 1). In the fully adjusted model, male sex and age (in years) were associated with higher risk of dying from an infectious disease or accident (Table 3). There were several risk factors for dying of a NCD, including self-reported history of heart diseases (HR = 2.25, 95% CI: 1.07–4.76; Table 4), and age in years (HR = 1.05, 95% CI: 1.01–1.09; Table 4).

**Table 3:** Risk factors for mortality of accidents and infectious diseases (*N* = 389)

Characteristic	Crude Model cHR (95% CI)	*p*-value	Model 1 aHR (95% CI)	*p*-value	Model 2 aHR (95% CI)	*p*-value
**Gender**						
Male	1		1		1	
Female	0.50 (0.28–0.91)	0.024	0.41 (0.22–0.79)	0.007	**0.37 (0.20–0.70)**	**0.002**
Age (years)	1.10 (1.07–1.13)	< 0.001	1.06 (1.02–1.10)	0.002	**1.06 (1.02–1.10)**	**0.001**
**Civil Status**						
Married or Cohabitation	1					
Single, Divorced or Widower	1.02 (0.55–1.90)	0.939				
**Education**						
Higher	1		1			
Secondary	0.78 (0.28–2.20)	0.643	0.55 (0.18–1.68)	0.294		
Primary	1.89 (0.76–4.65)	0.169	1.24 (0.49–3.14)	0.651		
No Education	3.20 (0.97–10.52)	0.055	1.55 (0.35–6.74)	0.562		
**Functional Parameters**						
Gait Speed (m/s)	0.06 (0.02–0.19)	< 0.001			0.28 (0.06–1.25)	0.097
Timed Up and Go test (sec)	1.08 (1.04–1.13)	< 0.001	1.08 (1.04–1.13)	< 0.001	1.04 (0.99–1.09)	0.158
**Clinical Variables**						
Abdominal perimeter	1.00 (0.98–1.02)	0.833				
Weight	0.98 (0.96–1.01)	0.172				
Malnutrition	0.83 (0.41–1.70)	0.614				
Obesity	0.58 (0.32–1.06)	0.079				
Dyslipidemia^a^	0.79 (0.43–1.44)	0.445				
Diabetes Mellitus^a^	0.87 (0.39–1.93)	0.738				
Arterial Hypertension^a^	1.42 (0.78–2.60)	0.255				
Heart Diseases^a^	1.67 (0.65–4.29)	0.291				
Stroke^a^	0.00 (0.00–0.00)^b^	< 0.001				
Rheumatological Diseases^a^	0.61 (0.31–1.21)	0.159				
Respiratory Diseases^a^	2.21 (0.88–5.56)	0.092				
Tuberculosis^a^	2.89 (0.72–11.56)	0.134				
Familial dysfunction^a^	2.11 (1.13–3.92)	0.019				
Sociofamily impairment	2.47 (1.18–5.18)	0.017			1.99 (0.91–4.36)	0.087
Cognitive impairment	1.67 (0.74–3.74)	0.216				
Insomnia^a^	1.23 (0.60–2.53)	0.569				
Depression	1.11 (0.54–2.32)	0.774				
Falls	1.58 (0.87–2.89)	0.134				
Edentulous	3.45 (0.83–14.30)	0.088				
Incontinence^a^	1.16 (0.58–2.30)	0.682				
Visual problem	0.86 (0.44–1.68)	0.652				
Hearing problem	1.27 (0.70–2.30)	0.441				
Polypharmacy	1.93 (1.06–3.52)	0.031			1.68 (0.90–3.14)	0.102
Tobacco Consumption	0.24 (0.03–1.71)	0.154				

Model 1 adjusted variables: age, sex, educational level and Timed Up and Go Test. Model 2 was developed with a nested forward multivariate Cox proportional hazard regression. cHR (crude hazard ratio), aHR (adjusted hazard ratio), 95% CI (95% confidence interval)

^a^, Diseases included in the table refer to self-reported history

^b^, Values were approximated (cHR = 1.63 × 10^−15^, 95% CI: 8.57 × 10^−16^-3.09 × 10^−15^). Estimates in bold are statistically significant at *p* < 0.05

**Table 4:** Risk factors for mortality of non-communicable diseases (*N* = 384)

Characteristic	Crude Model cHR (95% CI)	*p*-value	Model 1 aHR (95% CI)	*p*-value	Model 2 aHR (95% CI)	*p*-value
**Gender**						
Male	1		1		1	
Female	0.48 (0.25–0.91)	0.024	0.54 (0.28–1.05)	0.071	**0.50 (0.27–0.92)**	**0.027**
Age (years)	1.07 (1.04–1.11)	< 0.001	1.07 (1.03–1.11)	0.001	**1.05 (1.01–1.09)**	**0.026**
**Civil Status**						
Married or Cohabitation	1					
Single, Divorced or Widower	0.80 (0.41–1.59)	0.529				
**Education**						
Higher	1		1			
Secondary	0.89 (0.37–2.15)	0.800	0.79 (0.32–1.95)	0.606		
Primary	0.89 (0.37–2.14)	0.792	0.63 (0.27–1.49)	0.292		
No Education	1.01 (0.22–4.60)	0.993	0.53 (0.08–3.45)	0.510		
**Functional Parameters**						
Gait Speed (m/s)	0.31 (0.07–1.43)	0.133				
Timed Up and Go test (sec)	1.05 (1.01–1.10)	0.016	1.03 (0.98–1.09)	0.227	1.01 (0.96–1.06)	0.764
**Clinical Variables**						
Abdominal perimeter	1.02 (0.99–1.05)	0.139				
Weight	1.01 (0.99–1.03)	0.407				
Malnutrition	0.73 (0.33–1.59)	0.430				
Obesity	1.77 (0.78–4.03)	0.171				
Dyslipidemia^a^	0.85 (0.45–1.62)	0.618				
Diabetes Mellitus^a^	0.87 (0.36–2.08)	0.749				
Arterial Hypertension^a^	2.14 (1.08–4.26)	0.030			1.68 (0.85–3.33)	0.137
Heart Diseases^a^	3.01 (1.40–6.46)	0.005			**2.25 (1.07–4.76)**	**0.033**
Stroke^a^	0.77 (0.10–5.60)	0.792				
Rheumatological Diseases^a^	0.63 (0.30–1.29)	0.204				
Respiratory Diseases^a^	1.57 (0.47–5.27)	0.452				
Tuberculosis^a^	1.76 (0.25–12.68)	0.573				
Familial dysfunction^a^	0.57 (0.22–1.46)	0.243				
Sociofamily impairment	1.17 (0.60–2.26)	0.646				
Cognitive impairment	3.09 (1.56–6.11)	0.001			1.89 (0.90–3.96)	0.093
Insomnia^a^	1.43 (0.65–3.12)	0.371				
Depression	0.80 (0.34–1.92)	0.624				
Falls	0.95 (0.48–1.89)	0.891				
Edentulous	1.99 (0.61–6.51)	0.255				
Incontinence^a^	0.90 (0.41–1.97)	0.790				
Visual problem	1.44 (0.63–3.12)	0.386				
Hearing problem	1.76 (0.93–3.33)	0.084				
Polypharmacy	1.00 (0.52–1.90)	0.989				
Tobacco Consumption	1.43 (0.56–3.60)	0.452				

Model 1 adjusted variables: age, sex, educational level and Timed Up and Go Test. Model 2 was developed with a nested forward multivariate Cox proportional hazard regression. cHR (crude hazard ratio), aHR (adjusted hazard ratio), 95% CI (95% confidence interval)

^a^Diseases included in the table refer to self-reported history. Estimates in bold are statistically significant at p < 0.05

## Discussion

### Main results

In this population-based prospective cohort study of older people, and in the multivariate analysis, the Timed Up and Go test was a strong predictor of all-cause mortality, above and beyond other well-known risk factors like chronic diseases (e.g., diabetes). This suggests that the Timed Up and Go test could be part of all geriatric evaluations, in addition to the regular care and clinical assessment. Similarly, epidemiological studies could include this test in large population-based samples, to further understand its distribution and role to predict mortality. There were more risk factors strongly associated with NCDs mortality in comparison to mortality due to accidents and infectious diseases. This pinpoints the role of NCDs in the current epidemiological profile of older people living in resource-limited settings, growing apart form the idea that communicable diseases and accidents (e.g., falls) would be more relevant in these contexts.

Previous research has shown that mobility, equilibrium [44] and Timed Up and Go test scores are affected by advanced age [45]. The rise in the prevalence of medical and health conditions associated with the aging process will affect functional tests [46] and the Timed Up and Go test has the ability to reflect the burden of multimorbidity in different body systems that participate in coordination, mobility and balance [27]. The main mechanism suggested of the relationship of the increasing risk of mortality with an advancing age was through the development of multiple comorbidities that produce a poor physical performance [11].

Certainly, age is a variable that influences Timed Up and Go test and some reports have demonstrated that sex and BMI also affects Timed Up and Go test [5]. Nevertheless, Timed Up and Go test was an all-cause mortality predictor independent of age, which gives a window of opportunity for screening and intervention. While there is nothing we can do to stop aging, we could incorporate the Timed Up and Go test in the regular geriatric consultation, and intervene to improve the reasons for poor performance in this test.

### Results in context

The Timed Up and Go test was associated with higher risk of all-cause mortality. This goes in line with previous reports signalling that non-optimal results in the Timed Up and Go test increased the risk of all-cause mortality in the older population in high income countries by 20–60% [27, 47]; of note, these risk estimates would be higher when the underlying population had history of cardiovascular diseases or were women [29, 47]. The risk magnitude for all-cause mortality herein quantified was lower than that of these studies [27, 47], and there are potential explanations. First, these previous studies were conducted with a longer follow-up time contributing to detect more events (deaths) thus a stronger association. Second, we studied a population of Peru, a middle income country in Latin America where life expectancy increased into 2.3 years between 2005 and 2015 [48] compared with other countries [27, 47] where life expectancy increases just into 1.2–2.1 years in the same period [48].

The Timed Up and Go test is recommended as a routine screening test for falls [49], and its usefulness as a predictor of low physical performance and adverse events has been described [43, 47, 50]. It has been suggested that poor performance in the Timed Up and Go test is associated with higher mortality risk because it reflects underlying malaise, sarcopenia and chronic illness [27], all of which affects mobility, balance, strength and gait. The Timed Up and Go test is a more complex task that assess all these functions of mobility and strength, which could explain why it is a better mortality predictor than other features of the formal geriatric assessment [29]. Our results contribute and advance these recommendations by showing that the Timed Up and Go test is also associated with all-cause mortality. In so doing, we could suggest implementing the Timed Up and Go test as a regular screening test in older population, and not only to look for those at higher risk of falls.

Another important functional parameter in older population is the Gait Speed, and our results suggested, though with non-significant results in the adjusted models, that faster Gait Speed would reduce the risk of dying from communicable diseases and accidents. In the literature, poor Gait Speed has been associated with higher risk of all-cause mortality [51, 52], and it seems to be as good a tool as Timed Up and Go test to predict adverse events [43]. The reason because our results did not show a strong association could be lack of statistical power; small sample or few outcome events. In any case, our results provide preliminary evidence, pending further research, that the Gait Speed could also be incorporated as a standard screening test in older people care.

In our study, the Timed Up and Go test demonstrate to be a relevant mortality predictor, even independently of other socio-demographic traits and medical background. This functional test could be assessed more often in clinical evaluations and regular check-ups among older population in resource-limited countries. Future studies should assess the net benefit of including this test as standard and frequent care of the older population. Finally, talking about cause-specific mortality, history of heart diseases demonstrated to increase the risk for mortality of NCDs. Evidence showed that history of heart diseases increases the risk of cardiovascular disease associated with metabolic syndrome [53] and could contribute in this way to higher mortality risk due to NCDs.

### Strengths and limitations

This is a population-based prospective cohort study of people aged ≥60 in a resource-limited environment in Lima, Peru. Prospective research in gerontology, and in general addressing the wellbeing of older populations lack, particularly in low- and middle-income countries [31]. Our work contributed to this research field signalling the strong association with mortality of the Timed Up and Go test, above and beyond other stablished risk factors. All predictors at baseline were collected by trained geriatricians, and blood tests were analysed in one laboratory. Mortality information was based on death certificates, and not on reported information by a family member without further verification.

Nonetheless, there are limitations we acknowledge. First, although some information was collected by trained physicians with a standard questionnaire following a strict protocol, this information remains self-reported and could be biased (e.g., recall bias); this information would also depend on whether the participant is aware they have the condition or not. As we did for diabetes, hypertension and dyslipidaemia which were based on both self-reported and objective assessments, future work should verify our results with a more robust ascertainment of all self-reported predictors. Second, the number of outcome events was still limited to further inspect specific mortality causes (e.g., ischaemic heart disease versus stroke). Larger cohorts could provide this evidence, and future follow-ups of our cohort will also give lights about this. Additionally, hazard ratio values of stroke in Table 3 were too small, approximately zero. We believe this was a result of no stroke events in older adults who died from an infectious disease or accident; this estimate should not be interpreted as a significant finding. Third, confounding bias could influence the association between the functional parameters (Gait speed and Timed Up and Go tests) and mortality. Trying to minimize this bias, we considered multiple covariates as comorbidities, cognitive impairment, polypharmacy and visual problems in the analysis; however, residual confounding could not be ruled out due to inherent data. Fourth, we studied mortality, which is an extreme outcome. Unfortunately, it was not possible to use registry data to study other non-fatal (e.g., non-fatal myocardial infarction) and intermediate (e.g., emergency visits) outcomes; similarly, it was not possible to study healthy aging or functional decline. Future work, with an active face-to-face follow-up of the original participants could provide this information.

## Conclusions

The Timed Up and Go test was a strong all-cause mortality predictor, displacing other stablished risk factors like chronic diseases. This could support the recommendation to consistently include the Timed Up and Go test in all geriatric consultations. Likewise, this could suggest the introduction of this test in national and epidemiological large-scale surveys studying the wellbeing of the older people.

## Supplementary Information

**Additional file 1.** ICD-10 codes for Non-communicable disease and Infectious diseases or Accident cause of death.

## Data Availability

The datasets used and analysed during the current study are available from the corresponding author on reasonable request.

## Abbreviations

NCD: Non-Communicable Diseases
ICD-10: International Classification of Diseases
BMI: Body mass index
YLD: Years lived with disability
DALYs: Disability-adjusted life-year
