# Supplementary material for: Timed up and go test predicts mortality in older adults in Peru: a population-based cohort study
Source: BMC Geriatr. 2022 Jan 18;22:61. doi: 10.1186/s12877-022-02749-6 (PMC8767748; doi:10.1186/s12877-022-02749-6)
Supplement: Supplementary file 1 — Additional file 1. ICD-10 codes for Non-communicable disease and Infectious diseases or Accident cause of death. [file 12877_2022_2749_MOESM1_ESM.docx]

## Additional file 1. ICD-10 codes for Non-communicable disease and Infectious diseases or Accident cause of death

| Non-Communicable Disease | Infectious diseases or Accidents |
| --- | --- |
| ICD-10 C179 | ICD-10 A188 |
| ICD-10 C189 | ICD-10 A41.9 |
| ICD-10 C259 | ICD-10 A419 |
| ICD-10 C343 | ICD-10 J159 |
| ICD-10 C64 | ICD-10 J168 |
| ICD-10 C679 | ICD-10 J18.9 |
| ICD-10 D649 | ICD-10 J180 |
| ICD-10 E119 | ICD-10 189 |
| ICD-10 E14.9 | ICD-10 J47X |
| ICD-10 E141 | ICD-10 J690 |
| ICD-10 F028 | ICD-10 K650 |
| ICD-10 I10 | ICD-10 K83 |
| ICD-10 I10X | ICD-10 K830 |
| ICD-10 I219 | ICD-10 L899 |
| ICD-10 I48 | ICD-10 N390 |
| ICD-10 I50 | ICD-10 I469 |
| ICD-10 I619 | ICD-10 S069 |
| ICD-10 I64X |  |
| ICD-10 I678 |  |
| ICD-10 I679 |  |
| ICD-10 J449 |  |
| ICD-10 J841 |  |
| ICD-10 N189 |  |
| ICD-10 K74.6 |  |
